# Combining Pre-operative Diffusion Tensor Images and Intraoperative Magnetic Resonance Images in the Navigation Is Useful for Detecting White Matter Tracts During Glioma Surgery

**DOI:** 10.3389/fneur.2021.805952

**Published:** 2022-01-20

**Authors:** Manabu Tamura, Hiroyuki Kurihara, Taiichi Saito, Masayuki Nitta, Takashi Maruyama, Shunsuke Tsuzuki, Atsushi Fukui, Shunichi Koriyama, Takakazu Kawamata, Yoshihiro Muragaki

**Affiliations:** ^1^Faculty of Advanced Techno-Surgery, Institute of Advanced Biomedical Engineering and Science, Tokyo Women's Medical University, Tokyo, Japan; ^2^Department of Neurosurgery, Tokyo Women's Medical University, Tokyo, Japan

**Keywords:** diffusion tensor imaging, magnetic resonance imaging, fractional anisotropy, glioma, craniotomy

## Abstract

**Purpose:**

We developed a navigation system that superimposes the fractional anisotropy (FA) color map of pre-operative diffusion tensor imaging (DTI) and intraoperative magnetic resonance imaging (MRI). The current study aimed to investigate the usefulness of this system for neurophysiological monitoring and examination under awake craniotomy during tumor removal.

**Method:**

A total of 10 glioma patients (4 patients with right-side tumors; 5 men and 5 women; average age, 34 years) were evaluated. Among them, the tumor was localized to the frontal lobe, insular cortex, and parietal lobe in 8, 1, and 1 patient, respectively. There were 3 patients who underwent surgery on general anesthesia, while 7 patients underwent awake craniotomy. The index of DTI anisotropy taken pre-operatively (magnetic field: 3 tesla, 6 motion probing gradient directions) was analyzed as a color map (FA color map) and concurrently co-registered in the intraoperative MRI within the navigation. In addition to localization of the bipolar coagulator and the cortical stimulator for brain mapping on intraoperative MRI, the pre-operative FA color map was also concurrently integrated and displayed on the navigation monitor. This white matter nerve functional information was confirmed directly by using neurological examination and referring to the electrophysiological monitoring.

**Results:**

Intraoperative MRI, integrated pre-operative FA color map, and microscopic surgical view were displayed on one screen in all 10 patients, and white matter fibers including the pyramidal tract were displayed as a reference in blue. Regarding motor function, motor-evoked potential was monitored as appropriate in all cases, and removal was possible while directly confirming motor symptoms under awake craniotomy. Furthermore, the white matter fibers including the superior longitudinal fasciculus were displayed in green. Importantly, it was useful not only to localize the resection site, but to identify language-related, eye movement-related, and motor fibers at the electrical stimulation site. All motor and/or language white matter tracts were identified and visualized with the co-registration and then with an acceptable post-operative neurological outcome.

**Conclusion:**

Co-registering an intraoperative MR images and a pre-operative FA color map is a practical and useful method to predict the localization of critical white matter nerve functions intraoperatively in glioma surgery.

## Introduction

The usefulness of perioperative magnetic resonance imaging (MRI) in brain tumor resection has attracted research attention recently. In particular, intraoperative MRI for maximal tumor resection is aimed to prolong prognosis and reduce complications, making it an important method (1–5). Further, with the introduction of navigation-based tumor resection, MR images are now taken intraoperatively, and the position of the surgical tool held by the operator can be displayed on the MR image. The usefulness of this system as an intraoperative support device has been established, and it is now increasingly used in clinical practice. Similar MRI-guided navigation systems have been applied in facilities with no MRI systems inside the operating room, using MR images obtained the day before surgery. This system provides more information and allows a more accurate surgery than that performed only based on the anatomical structure on pre-operative MRI, making it an important surgical support strategy (6).

Advances in pre-operative MRI have made it possible to obtain more detailed functional information of brain tumors and surrounding brain tissues. The acquisition of functional information on brain tumors and surrounding brain tissues has recently attracted attention as a possible method for reducing perioperative functional complications. High-field MRI devices have enabled acquisition of pre-operative MRI function images useful for predicting brain function sites during surgery and estimating brain function after tumor resection. Functional images obtained from pre-operative MRI can be broadly divided into (1) functional MRI (fMRI) and (2) diffusion tensor imaging (DTI) (7). In fMRI, language tasks are performed to image language-dominant hemispheres and functional localization (8). Meanwhile, DTI enables imaging of white matter fibers involved in motor and language functions (FA color map) as well as the tractography of white matter tracts (9). However, there have been reports that notable white matter tracts were not visualized (false negatives) on perioperative tractography (10, 11).

We believe that this issue will be resolved by directly confirming brain function during surgery. Functional mapping under awake craniotomy allows the surgeon to clearly define language, positive motor and negative motor areas as well as the positions of white matter fibers connected with speech and motor functions, helping to prevent unexpected neurological deficits. A meta-analysis demonstrated late severe neurological deficit in 3.4% of patients who underwent resection with stimulation mapping, compared to 8.3% of patients who underwent resection without mapping (12). For intraoperative confirmation of brain function, including the white matter of the cerebral cortex, it is important to capture longitudinal changes in motor and language functions before and after the start of tumor resection. Assessing motor-evoked potential (MEP) with the cerebral cortex indwelling strip is also helpful. Brain function during awake craniotomy is more accurate than that determined on pre-operative imaging. However, frequent brain stimulation can cause seizures, and there are issues with the accuracy of MEP measurement.

Therefore, the optimal strategy for achieving maximal tumor resection and reducing neurological complications is needed. We have previously developed a navigation system that superimposes the FA color map of pre-operative DTI and intraoperative MRI (13). In addition to predicting the FA color map display of DTI as a pre-operative white matter functional site, our system can be used to evaluate brain function complementary to neurophysiological examinations and direct neurological function evaluation under awake craniotomy. The current study aimed to investigate the usefulness of this system for neurophysiological monitoring and examination under awake craniotomy during tumor removal.

## Methods

Among patients who underwent brain tumor resections after April 2016, 10 consecutive patients (5 males, 5 females; average age, 34 years) with co-registered pre-operative DTI and intraoperative MRI were evaluated. Among them, 8 and 2 patients underwent their first and second surgeries, respectively (Table 1). There were 4 and 6 patients with right- and left-sided tumors. The tumors were located in the frontal lobe in 8 patients; insular cortex, 1 patient; and parietal lobe, 1 patient. Surgery was performed under general anesthesia and awake craniotomy in 3 and 7 patients, respectively.

**Table 1:** Patient characteristics and clinical results with image integration of pre-operative DTI-FA color map and intraoperative MRI.

**Case**	**Age, sex**	**Pathology, WHO 2016 grade**	**Initial or additional**	**Side-location**	**Anesthesia**	**Object for**	**Clinical results (tumor removal rate and symptoms)**		
						**DTI fusion**	**R. rate**	**Post-ope within 7 days**	**Post-ope at 3 months later**
1	19, M	AA, 3	Initial	Rt-Insula	General	M	90	None	None
2	36, M	Oligo, 2	Initial	Lt-Frontal	Awake	M, B	98	Paresis (u-l, 4/5), Aphasia	Paresis (u-l, 4/5), Aphasia
3	37, F	AA, 3	Initial	Lt-Frontal	Awake	M, B	94	Paralysis (u, 0/5), Aphasia	Paresis (u, 4/5)
4	43, M	AA, 3	Initial	Rt-Frontal	Awake	M, B	95	Paresis (u, 2–3/5)	Paresis (u, 4/5)
5	37, F	AA, 3	Additional	Lt-Parietal	Awake	M, B, W	95	Mild agnosia	None
6	24, F	GBM, 4	Initial	Rt-Frontal	Awake	M	95	Paresis (u-1/5 and l-3/5)	Paresis (u-l, 4/5)
7	41, F	AA, 3	Additional	Rt-Frontal	General	M	95	Paresis (u-l, 4/5), Dysarthria	Dysarthria
8	29, F	GBM, 4	Initial	Lt-Frontal	General	M	90	Paresis (u-l, 1/5), Aphasia	Paresis (u-2/5 and l-4/5), Aphasia
9	32, M	AA, 3	Initial	Lt-Frontal	Awake	M, B	75	Paresis (u-l, 3–4/5), Aphasia	Paresis (u-l, 4/5)
10	41, M	AO, 3	Initial	Lt-Frontal	Awake	M, B	70	Paresis (u-l, 4/5), Dysarthria	None

*AA, Anaplastic astrocytoma; Oligo, Oligodendroglioma; GBM, Glioblastoma; AO, Anaplastic oligodendroglioma; Rt, Right; Lt, Left, Object for DTI fusion (B, frontal language; W, posterior language; M, motor-sensory); R. rate, Tumor removal rate (%)*.

*Motor symptom (paralysis/paresis) was evaluated by Manual Muscle Test (1–5 scale) using u-l (upper limb and lower limb), u (upper limb) and l (lower limb)*.

**Figure 1:**
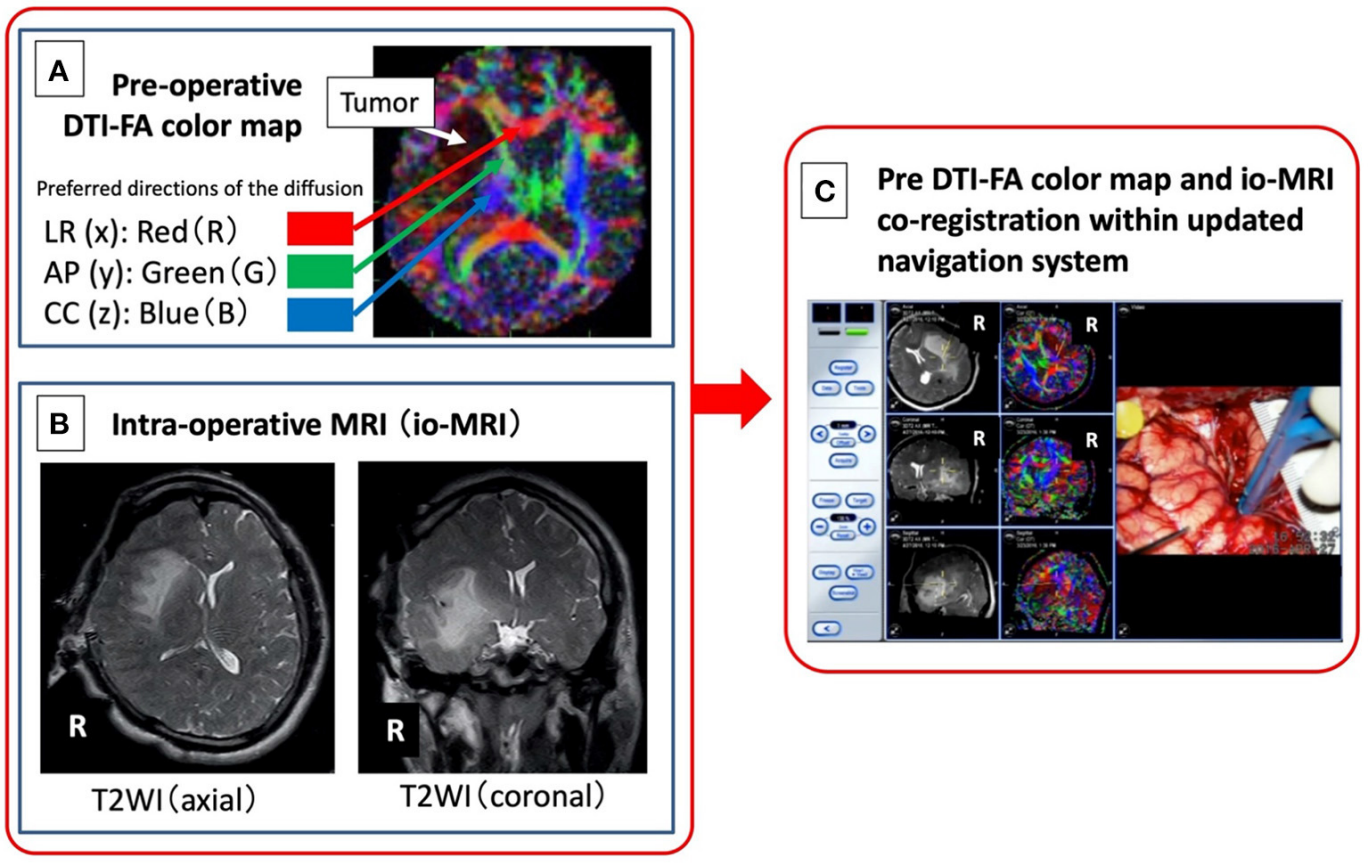
Image integration of pre-operative DTI-FA color map **(A)** and intraoperative MRI **(B)** navigation system. A newly developed system **(C)** in which an FA color map, as an objective measure of neural function, from pre-operative DTI is combined with intraoperative MRI image obtained after craniotomy, is developed.

Pre-operative DT images were used during surgery to focus on motor fibers near the tumor in all 10 patients and to language fibers in 6 patients. Pre-operative DT images were acquired using a 3-Tesla magnetic field MRI (Philips ACHIEVA™) with 6 directions of diffusion sensitizing gradient and nearly isotropic voxel size. Raw DTI data obtained in the MRI workstation was color mapped (FA color map) *via* RGB conversion for the index of anisotropy. The left and right x directions were displayed in red (R), the front and back y directions were displayed in green (G), and the up and down z directions were displayed in blue (B) according to the same coordinate axes as in normal MRI (see Figure 1A). The FA color map images acquired as 70 consecutive DICOM images in the axial plane and 128 consecutive DICOM images in the coronal plane enabled prediction of the relationship between tumor position and white matter fibers as pre-operative DTI.

Intraoperative MR images (magnetic field strength of 0.3 Tesla, Hitachi™), including 100 T1- and T2-weighted images, obtained after craniotomy (Figure 1B) was read on a navigation system (BrainLAB™). Next, the DICOM image of the FA color map created from the pre-operative DTI (Figure 1A) was read on the same navigation system, and the image is co-registered with the previous intraoperative MRI and displayed concurrently (Figure 1C). Finally, a bipolar coagulator and an electrical stimulation probe (manufactured by Unique Medical™) were registered using a sterile antenna device and a skull-fixed marker (14).

With this method, the position information of the bipolar coagulator and the electrical stimulation probe operated by the operator was displayed simultaneously on the intraoperative MRI and the pre-operative DTI (FA color map). For functional evaluation of motor white matter fibers, MEP findings obtained from the scalp indwelling needle and cerebral cortex surface strip prepared at the start of surgery, direct patient movement confirmation (under awake craniotomy), and FA color map display on the navigation system were used. For the functional evaluation of verbal white matter fibers, the language function of awake patients was evaluated using the language function test system [Intraoperative Examination Monitor for Awake Surgery, IEMAS (15, 16)] we developed in 2004. In addition to direct confirmation and video recording, nerve position was confirmed using the FA color map display on the navigation system.

All procedures performed in studies involving patients were in accordance with the ethical standards of the ethics committee of Tokyo Women's Medical University and with the 1964 Declaration of Helsinki, as revised in 2013. Each patient provided informed consent before the surgical procedure.

## Results

In all 10 patients, intraoperative MRI, FA color map, and intraoperative field microscope images were displayed in real time along with the position of the bipolar and probe on the same screen of the navigation monitor. Focusing on the white matter fibers represented by the pyramidal tract, the part that can be expected to run the motor nerves of the lower limbs was displayed in real time in blue (vertical direction). Thus, navigation information other than the tumor position was obtained. In addition to navigation information, resection was confirmed with regular MEP monitoring in all 10 patients. In patients under awake craniotomy, efforts were made to reduce complications while directly evaluating motor function. The language-related nerve tract (arcuate fasciculus) was adequately displayed in green (anterior-posterior direction), providing the surgeon real-time navigation information for the monitoring of verbal symptoms.

All 7 patients who underwent awake surgery received IEMAS assessment for confirmation of language function. Six patients developed motor paralysis (Manual Muscle Test evaluation) at 3 months after the tumor resection; of them, 2 patients also developed expressive aphasia. In the 4 patients, 1 and 3 patients had residual dysarthria and no motor/language function complications, respectively. The average tumor resection rate was 89.7% (70–98%). Histopathological findings (World Health Organization grade) were grade 2 in 1 patient, grade 3 in 7 patients, and grade 4 in 2 patients (see Table 1).

**Figure 2:**
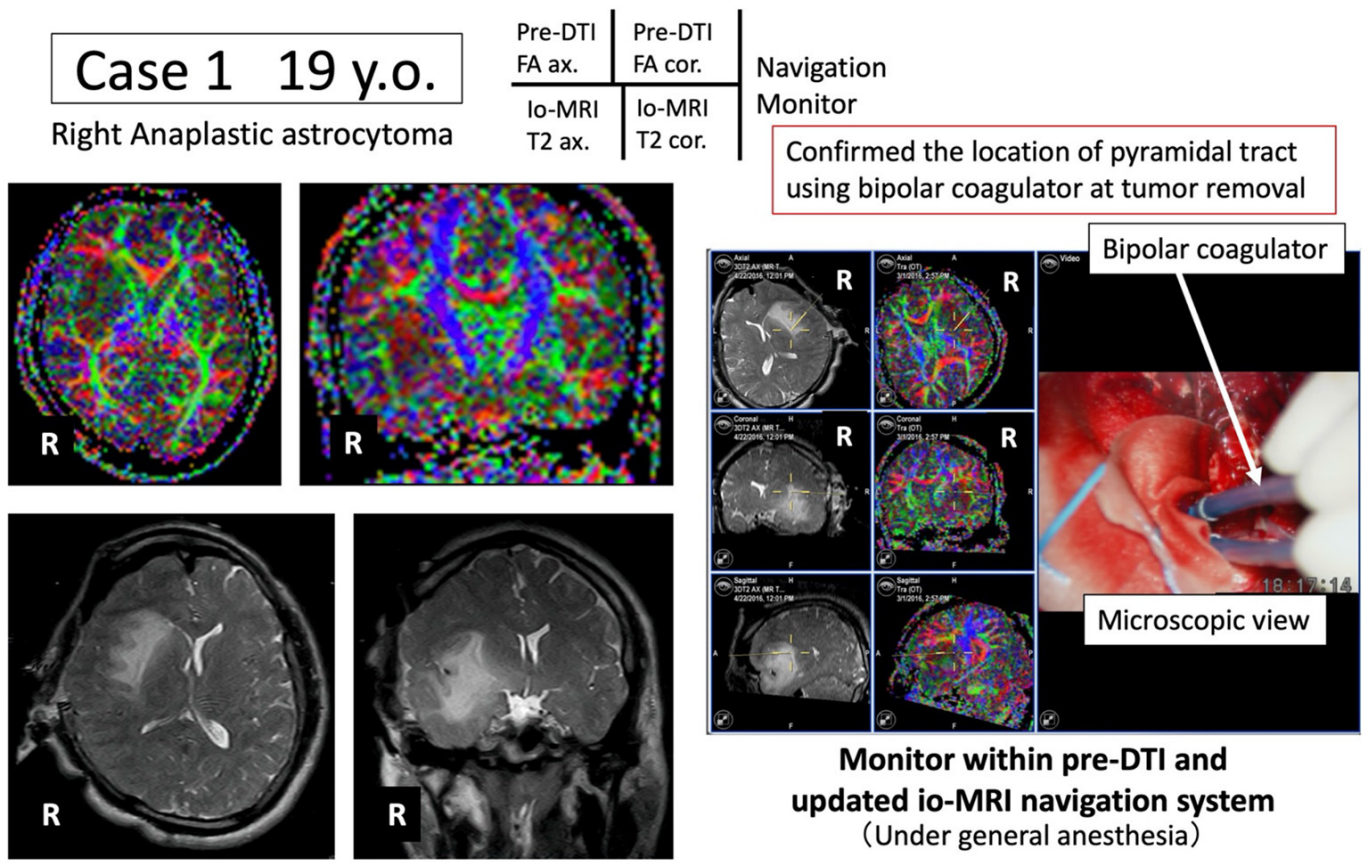
Case 1: navigation based on pre-operative pyramidal tract motor function information. Under general anesthesia, tumor resection is advanced while the pyramidal tract (blue) is monitored in real time using the newly developed navigation system.

**Figure 3:**
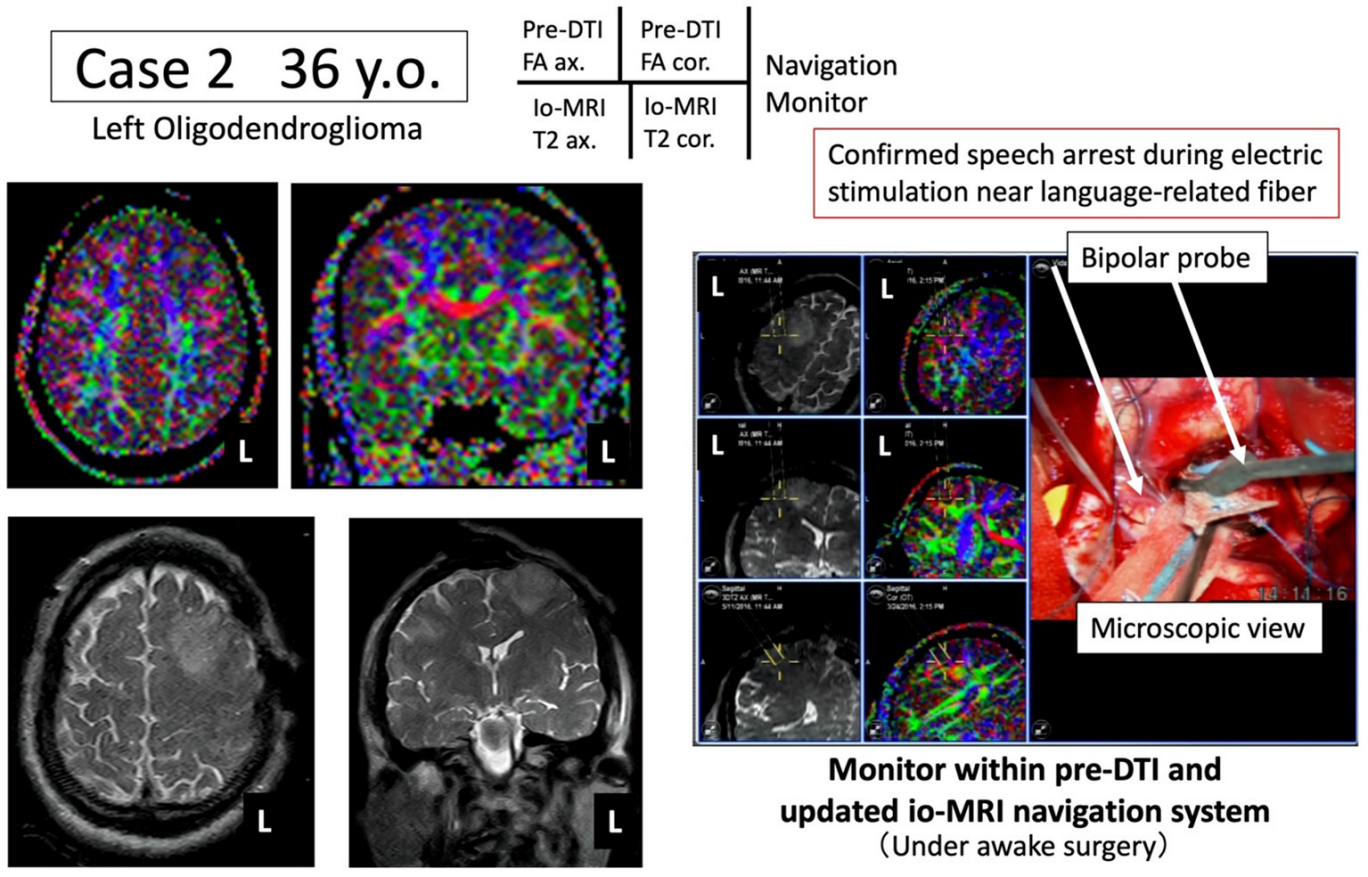
Case 2: navigation based on pre-operative arcuate fasciculus language function information. Under awake craniotomy, tumor resection is performed while the arcuate fasciculus (green) is monitored in real time using the newly developed navigation system.

**Figure 4:**
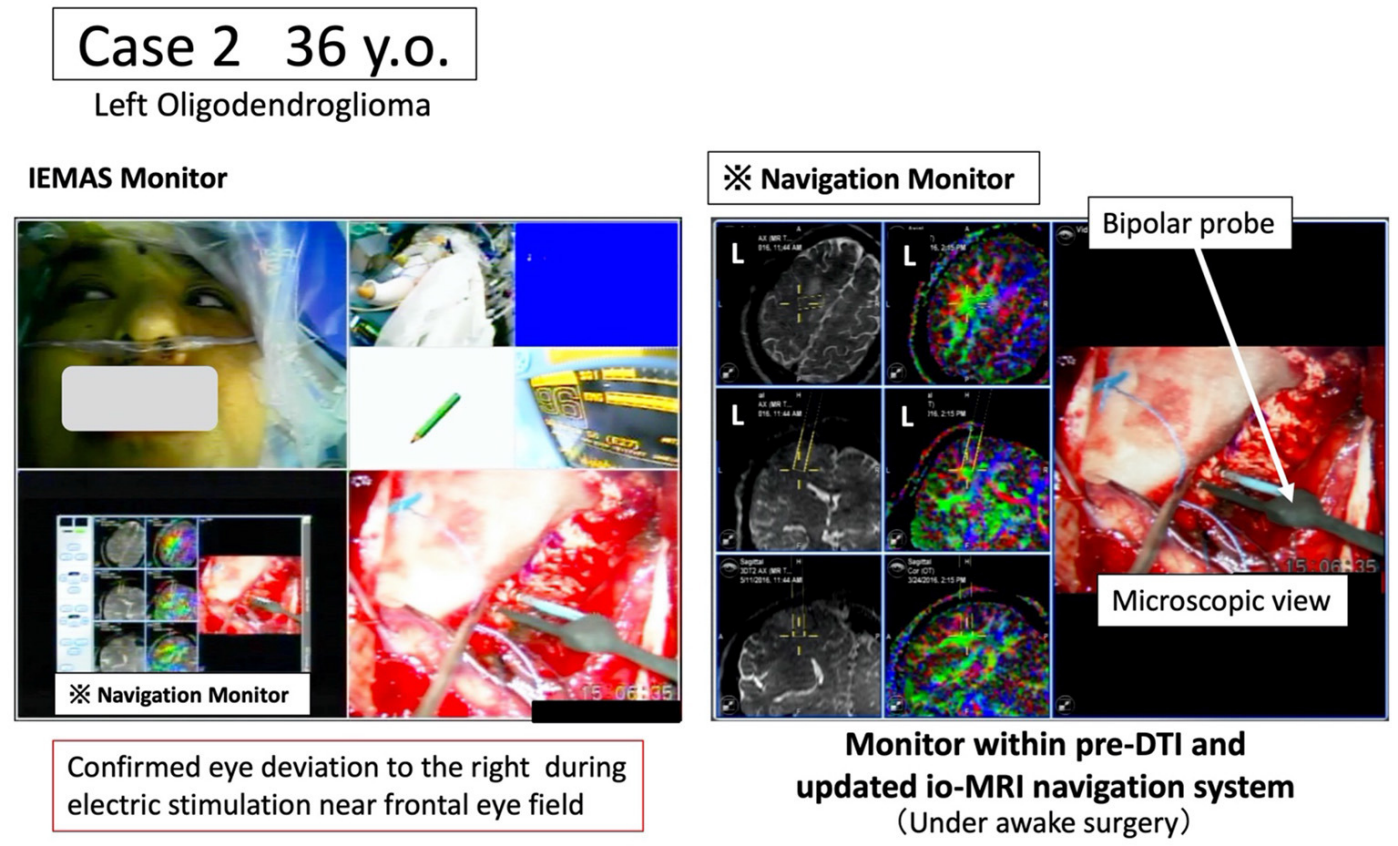
Case 2: pre-operative arcuate fasciculus in which the joint eye movement center is identified based on language function information and language function tests. Under awake craniotomy, the tumor resection is performed while the white matter fiber (green) related to the joint eye movement center is monitored in real time using the newly developed navigation system. The intraoperative language monitoring system (IEMAS) is also used concurrently, and the language reaction associated with the presentation of the language image task is determined by both the surgeon and the examiner, making the system highly useful for awake surgery.

We present representative cases below. The position of the tumor and surrounding white matter tracts was more accurately indicated by the navigation system. The surgeon can now refer to the pre-operative DTI-FA color map that is displayed in real time in conjunction with the intraoperative MRI image, making it possible to reduce the risk (see Figures 2, 3). Furthermore, when a language function test was performed near the arcuate fasciculus of the white matter of the frontal lobe, a task response was not possible due to joint eye deviation during electrical stimulation mapping, and a false positive mapping condition was confirmed (Figure 4). In particular, it was shown that the pre-operative DTI-FA color map provides useful information for identifying motor fibers, language-related fibers, and eye movement-related fibers corresponding to bipolar and electrical stimulation probe positions during awake surgery.

### Illustrative Cases

#### Case 1: General Anesthesia, Confirmation of Motor Nerves

A 19-year-old male underwent tumor resection under general anesthesia for Grade 3 glioma of the right insular cortex. The pre-operative DTI-FA color map indicated that the pyramidal tract (indicated by blue) was close to the tumor. By referring to the FA color map image co-registered with the intraoperative MRI updated after craniotomy, the tumor was successfully resected while visualizing the pyramidal tract in real time (Figure 2). Post-operative complications were also avoided while monitoring MEP findings.

#### Case 2: Awake Craniotomy, Confirmation of Nerve Involved in Joint Eye Movements and Verbal Response

A 36-year-old male underwent tumor resection *via* awake surgery for grade 2 glioma of the left frontal lobe. The pre-operative DTI-FA color map indicated that the pyramidal tract (indicated by blue) and the arcuate fasciculus (superior longitudinal fasciculus) (indicated by green) were close to the tumor. A language function test performed during tumor resection in the deep white matter showed a speech arrest associated with electrical stimulation. This was consistent with the FA color map findings (green) of the white matter fibers predicted to be the superior longitudinal fasciculus (Figure 3, positive findings of language mapping). Meanwhile, language function test performed on further tumor resection confirmed that the response to the language task was not possible because joint eye deviation was induced during white matter electrical stimulation (false-positive findings of language mapping, Figure 4). We were able to obtain useful findings for anatomical identification of white matter tracts while accurately recording the nerve responses involved in joint eye movement. By referring to the FA color map image co-registered with the intraoperative MRI updated after craniotomy, it was possible to excise the tumor while visualizing the pyramidal tract and arcuate fasciculus (superior longitudinal fasciculus) in real-time during monitoring of MEP and IEMAS findings. This help reduced post-operative complications.

## Discussion

Intraoperative functional evaluation of the brain is important in glioma surgery, but current methods have few information to localize critical white matter nerve with limited accuracy. In this study, a newly developed system in which an FA color map, as an objective measure of neural function, from pre-operative DTI is combined with intraoperative MRI image obtained after craniotomy, is developed and evaluated as a clinical assistance to contribute glioma surgery with an acceptable accuracy.

Differences in pre- and intraoperative MRI findings, which mainly occur from mechanical error due to navigation and intraoperative brain shift of post-craniotomy brain tissue, are important factors that need to be considered in brain surgery (17). Particularly, many navigation errors have been evaluated in intraoperative MR images in neurosurgery. For example, in navigating and registering the marker to be attached to the patient during MRI, the skin marker attached to the scalp or face produces an error of 1.5–2 mm (maximum, 4.2 mm) (18). Intraoperatively, driving a marker into the skull after craniotomy (born marker) leads to an error of 0.84–1.4 mm (19). Pre-operative MRI also has issues of position error (brain shift) due to intraoperative changes (20). This is a phenomenon in which the brain including lesions sinks more than before surgery after cerebrospinal fluid outflow or tumor removal (21). Importantly, it is difficult to predict, although it is affected by the size of the craniotomy, tumor histology, patient position, presence/absence of ventricular opening, and amount of excised tumor.

The post method (22) and intraoperative imaging after brain shift are useful methods to address this problem (23, 24). Although navigation errors are difficult to identify intraoperatively, it is possible to infer mechanical errors by confirming the marker position and errors due to brain shift by confirming the position of the brain surface [corresponding to (1) in Table 2]. Intraoperative MRI was first performed in 1993 by Black et al. (25) for brain tumor resection in the United States. In Germany, intraoperative imaging is mainly used in neurosurgery. The main purpose of intraoperative MRI is to safely remove lesions in challenging locations, such as tumors existing in parenchymal organs. It can confirm the presence of residual lesions and determine the need to extend the resection, ultimately improving the tumor resection rate.

**Table 2:** Summary of a combination of image co-registration within navigation system in OR.

	**Pre-operative**	**Intra-operative**		**DWI or DTI image co-registration**	
	**DTI**	**io-DWI**	**io-DTI**	**Advantage**	**Disadvantage**
Pre-operative MRI	(1)	–	–	No need for MR in OR	Large brain shift effect
io-MRI (Middle F)	–	(2)	–	Small brain shift effect	Limited to pyramidal tract in DWI
io-MRI (Middle F)	Current study (3)	–	–	Practical, Informative	Middle brain shift effect
io-MRI (High F)	–	–	(4)	Small brain shift effect	Long time for MR scan and setting

*Middle F, Middle field magnetic strength (0.3–0.4 Tesla); High F, High field magnetic strength (1.5–3.0 Tesla); io, intra-operative; DTI, Diffusion Tensor Image; DWI, Diffusion Weighted Image; OR, Operation Room*.

Intraoperative MR images are usually acquired after craniotomy, and the MRI image is registered in the operating room navigation system. This system can display the position of the surgeon's surgical instrument on the MR image, making it particularly useful. Intraoperative MR images are updated imaged reflecting the brain shift from the parenchymal position of the brain due to craniotomy. They are more accurate for surgical navigation than pre-operative MRI. Although navigation error cannot be avoided, the surgery can perform more quickly and precisely.

This system has been used for 20 years in Japan. Medium-low magnetic field MRI is sufficient to control the removal of brain tumors (particularly gliomas), which is a major role of intraoperative MRI (26, 27). Both the magnetic field strength and the system design and operation are important. Medium magnetic field with enhanced functionality can depict the pyramidal tract using an intraoperative diffusion weighted image (DWI) image (28) [corresponding to (2) in Table 2]. We registered 0.3T intraoperative DWI and MRI images in the intraoperative navigation system for functional evaluation of the pyramidal tract nerve, and this allowed us to obtain functional images that minimized the effects of intraoperative brain shift. In addition, we used the FA color maps obtained pre-operatively using DTI [corresponding to (3) in Table 2].

There have been profound advances in visualization of brain white matter, but there are also reports that results differ according to the MRI method, software for visualization, and the purpose of the analysis. There are cases where white matter tracts are not visualized (false negatives) and other excess white matter tracts are visualized (false positives) (11). To solve this problem, we used FA color maps as objective brain function measures and included these in the navigation system along with intraoperative MR images. This enabled real-time and concurrent visualization of the surgery and brain function, providing useful information. It provides the surgeon with real-time information on the white matter tracts related to motor and verbal functions, reducing perioperative complications. Furthermore, false-positive intraoperative language impairments were identified through stimulation of the joint eye movement center, contributing to achieving adequate tumor resection. Collectively, these results support that when pre-operative DTI and intraoperative MRI are combined within an allowable error range, they can be useful as a navigation system and obtain information on white matter function [corresponding to (3) in Table 2].

Intraoperative DTI imaging can now be performed simultaneously with intraoperative MRI. fMRI and DTI need to be acquired with a high-magnetic-field MRI device, and brain shift can be minimized by combining intraoperative DTI with intraoperative MRI using a high-magnetic-field MR device [corresponding to (4) in Table 2]. Intraoperative MRI is now widely used, and image-guided navigation systems that provide functional information about the brain have become increasingly important and more accurate. These systems improve the safety of surgery by providing reliable and useful information, ultimately improving prognosis by increasing the degree of intraoperative resection. As shown in Table 2, between (1) the conventional combination method of image co-registration within navigation system with only pre-operative DTI without intraoperative MRI and (4) the ideal method using intraoperative DTI + intraoperative MRI, there is our new technique (3) pre-operative DTI + intraoperative MRI. This (3) is practical and best choice to use at our operating room with a middle-field MRI device.

This study has some limitations. First, as mentioned in the comparison between (3) and (4) in Table 2, DTI imaging that minimizes the effect of brain shift requires high-field MRI in the operating room. Since our environment operated by low-field MRI and the operating room environment are different, it is impossible to evaluate white matter nerve function based on a unified standard. When a high-magnetic-field MRI device is used intraoperatively, it is necessary to examine and verify the improvement of image artifacts and confirm image accuracy, intraoperative acquisition time, and preparation for DTI FA color map creation. Next, the accuracy of the intraoperative MRI images of the high magnetic field and the low magnetic field is compared, and then the co-registration of the high magnetic field MRI and the pre-operative DTI is performed on the navigation to compare the accuracy. Ultimately, we believe that a procedure aiming for clinical application that simultaneously utilizes intraoperative MRI and intraoperative DTI in a high magnetic field is necessary. Third, we do not have the information that can describe the accuracy of this technique, but we are thinking of a method to evaluate the impact of the new method on the conventional method. In other words, a prospective comparative study was conducted between the group that performed the conventional method and the group that added the proposed method to the conventional method. We would like to conduct clinical research with the endpoints of (1) brain function prognosis, degree of rehabilitation, (2) tumor removal rate, and degree of survival prognosis.

## Conclusion

Co-registering intraoperative MR images with pre-operative FA color maps is a useful and practical method for intraoperative localization of critical white matter nerves and functional assessment in glioma surgery.

## Data Availability Statement

The original contributions presented in the study are included in the article/supplementary material, further inquiries can be directed to the corresponding author/s.

## Ethics Statement

The studies involving human participants were reviewed and approved by the Ethics Committee of Tokyo Women's Medical University. The patients/participants provided their written informed consent to participate in this study.

## Author Contributions

YM, MT, TM, and TK: conception and design. MT, HK, TS, MN, ST, AF, and SK: acquisition of data. YM and MT: analysis and interpretation of data. MT: drafting the article. YM, MT, HK, TS, MN, TM, ST, AF, SK, and TK: reviewed submitted version of manuscript. YM: approved the final version of the manuscript behalf of all authors. YM and TK: study supervision. All authors contributed to the article and approved the submitted version.

## Funding

This research was supported by JSPS Grant-in-Aid for Scientific Research (Grant Number C-19K12845), NICT (National Institute of Information and Communications, Grant Number 22009) and AMED (Grant Number JP21he1602003).

## Conflict of Interest

The authors declare that the research was conducted in the absence of any commercial or financial relationships that could be construed as a potential conflict of interest.

## Publisher's Note

All claims expressed in this article are solely those of the authors and do not necessarily represent those of their affiliated organizations, or those of the publisher, the editors and the reviewers. Any product that may be evaluated in this article, or claim that may be made by its manufacturer, is not guaranteed or endorsed by the publisher.
